# MEPE-Derived ASARM Peptide Inhibits Odontogenic Differentiation of Dental Pulp Stem Cells and Impairs Mineralization in Tooth Models of X-Linked Hypophosphatemia

**DOI:** 10.1371/journal.pone.0056749

**Published:** 2013-02-22

**Authors:** Benjamin Salmon, Claire Bardet, Mayssam Khaddam, Jiar Naji, Benjamin R. Coyac, Brigitte Baroukh, Franck Letourneur, Julie Lesieur, Franck Decup, Dominique Le Denmat, Antonino Nicoletti, Anne Poliard, Peter S. Rowe, Eric Huet, Sibylle Opsahl Vital, Agnès Linglart, Marc D. McKee, Catherine Chaussain

**Affiliations:** 1 EA 2496, Pathologies, Imaging and Biotherapies of the Tooth, UFR Odontologie, University Paris Descartes PRES Sorbonne Paris Cité, Montrouge, France; 2 AP-HP Odontology Department Bretonneau – Louis Mourier, Hôpitaux Universitaires Paris Nord Val de Seine, Paris France; 3 Centre de Référence des Maladies Rares du Métabolisme du Phosphore et du Calcium, AP-HP, Kremlin Bicêtre, France; 4 Institut Cochin, University Paris Descartes PRES Sorbonne Paris Cité, Paris, France; 5 The Kidney Institute, University of Kansas Medical Center, Kansas City, Kansas, United States of America; 6 AP-HP Odontology Department Charles Foix, Ivry Sur Seine, France; 7 Université Paris-Est, Laboratoire CRRET, CNRS, Créteil, France; 8 APHP Endocrinology and Diabetology for Children, Bicêtre Paris Sud Hospital, Kremlin Bicêtre, France; 9 Université Paris-Sud, Kremlin Bicêtre, France; 10 Faculty of Dentistry, and Department of Anatomy and Cell Biology, McGill University, Montreal, Quebec, Canada; 11 Inserm UMRS698, Paris, France; 12 Denis Diderot University, UMRS698, Paris, France; Instituto Butantan, Brazil

## Abstract

Mutations in *PHEX* (phosphate-regulating gene with homologies to endopeptidases on the X-chromosome) cause X-linked familial hypophosphatemic rickets (XLH), a disorder having severe bone and tooth dentin mineralization defects. The absence of functional PHEX leads to abnormal accumulation of ASARM (acidic serine- and aspartate-rich motif) peptide − a substrate for PHEX and a strong inhibitor of mineralization − derived from MEPE (matrix extracellular phosphoglycoprotein) and other matrix proteins. MEPE-derived ASARM peptide accumulates in tooth dentin of XLH patients where it may impair dentinogenesis. Here, we investigated the effects of ASARM peptides *in vitro* and *in vivo* on odontoblast differentiation and matrix mineralization. Dental pulp stem cells from human exfoliated deciduous teeth (SHEDs) were seeded into a 3D collagen scaffold, and induced towards odontogenic differentiation. Cultures were treated with synthetic ASARM peptides (phosphorylated and nonphosphorylated) derived from the human MEPE sequence. Phosphorylated ASARM peptide inhibited SHED differentiation *in vitro*, with no mineralized nodule formation, decreased odontoblast marker expression, and upregulated MEPE expression. Phosphorylated ASARM peptide implanted in a rat molar pulp injury model impaired reparative dentin formation and mineralization, with increased MEPE immunohistochemical staining. In conclusion, using complementary models to study tooth dentin defects observed in XLH, we demonstrate that the MEPE-derived ASARM peptide inhibits both odontogenic differentiation and matrix mineralization, while increasing MEPE expression. These results contribute to a partial mechanistic explanation of XLH pathogenesis: direct inhibition of mineralization by ASARM peptide leads to the mineralization defects in XLH teeth. This process appears to be positively reinforced by the increased MEPE expression induced by ASARM. The MEPE-ASARM system can therefore be considered as a potential therapeutic target.

## Introduction

Mineralization is regulated by a number of determinants, including small molecules such as pyrophosphate, and proteins (including enzymes and hormones), that act either locally in the extracellular matrix and/or systemically to regulate calcium and phosphate metabolism. Currently, the complex links between these molecules are not well understood. Of note for tooth dentin (unlike bone) is that dentin is not involved in the regulation of the calcium and phosphate metabolism, and dentin is not remodeled. Thus, it is a tissue showing a permanent chronological record of disease that may be useful in deciphering these complex molecular interactions and mechanisms of mineralization.

Skeletal mineralization is severely impaired in patients with mutations in the genes encoding mineralization-regulating proteins [1]. Genetic disorders, such as X-linked familial hypophosphatemic rickets (XLH), may affect both bone and tooth mineralization [2]. Mineralization defects in XLH appear in children as rickets with severe skeletal deformities and as osteomalacia and bone or join pain in adults. Patients with XLH frequently present spontaneous dental abscesses, without any history of tooth trauma or decay [3], [4], [5]. Despite the fact that teeth of patients with XLH seem visibly normal, radiography commonly reveals thin enamel and radiolucent dentin – the latter being associated with unusually large pulp chambers. In XLH, dentin mineralization is impaired [6], [7], [8], with the defect appearing as unmerged, persistent mineralization foci (calcospherites) surrounded by extensive interglobular regions of unmineralized dentin matrix [6]. These abnormalities may lead to rapid pulp necrosis associated with periapical bone infection. Within the pathologic, unmineralized interglobular dentin matrix, many of the extracellular matrix (ECM) components, including noncollagenous proteins (NCPs), are degraded [7], [9], [10], [11]. Among them, degraded members of the SIBLING family (small integrin-binding ligand N-linked glycoproteins) have been identified in XLH dentin [12], [13]. This family of proteins shares a common acidic serine- and aspartate-rich motif (ASARM), which has a key role in the mineralization process [14], [15], [16], [17], [18], [19].

Among the SIBLING proteins, MEPE (matrix extracellular phosphoglycoprotein) has been shown to be involved in pathologic processes associated with hypophosphatemic rickets [16], [20], [21], [22], [23]. MEPE is expressed by both osteoblasts and odontoblasts and is normally protected from cleavage by a protein-protein interaction between its ASARM motif and PHEX (phosphate-regulating gene with homologies to endopeptidases on the X-chromosome) [24], [25] − a transmembrane endopeptidase. More than 80% of patients with familial hypophosphatemic rickets carry a mutation in the *PHEX* gene [26], [27], [28], [29]. In these patients, unprotected MEPE is exposed to pathologic cleavage by local proteinases such as cathepsin B, releasing ASARM peptides into the ECM and the circulation [24], [30], [31]. Moreover, since ASARM is normally a substrate for the enzymatic activity of PHEX [32], [33], the lack of functional PHEX in XLH patients results in the accumulation of these proteinase-resistant peptides that are thought to lead to mineralization defects in bone and tooth ECM [12], [14], [23], [34]. Other mineralization-regulating SIBLING proteins, such as OPN and DMP1 (dentin matrix protein 1), as well as the ASARM-containing peptides derived from their cleavage, may also be involved in the mineralization pathology [14], [32], [35], [36]. Previous *in vitro* studies have shown that mouse-derived bone marrow stromal cells (BMSCs) and osteoblasts treated with phosphorylated MEPE-derived (and OPN-derived) ASARM synthetic peptides failed to properly mineralize their ECM [14], [32], [33], [37], [38].Since human teeth are severely affected by the disease [3], [6], this study aimed to investigate *in vitro* and *in vivo* the effects of the MEPE-derived ASARM peptide on tooth dentin mineralization. We used pulp progenitor stem cells from human exfoliated deciduous teeth (SHEDs), as we and others have shown that deciduous teeth are mostly affected in patients with XLH [3], [7], [8], [30]. These cells were induced toward an odontogenic differentiation program using a cell culture collagen/tooth slice 3D scaffold model. In parallel, we implanted MEPE-derived ASARM peptides into surgically injured pulp of rat molars [39], and their effects on reparative dentin formation were evaluated. From these *in vitro* and *in vivo* studies reported here, we demonstrate that phosphorylated MEPE-derived ASARM peptide inhibits dentin mineralization, disturbs odontoblast differentiation and dramatically upregulates MEPE expression. This ASARM peptide − previously identified and shown to accumulate in dentin from patients with XLH [12] − therefore appears to be a key molecule in the pathogenesis of tooth dentin abnormalities as observed in XLH patients.

## Materials and Methods

### Human Teeth

Teeth were obtained from the Dental Department of Hopitaux Universitaires Paris Nord Val de Seine, AP-HP, France. Deciduous teeth were collected after trauma or after exfoliation from three healthy young children (3–7 years of age). Permanent third molars were obtained after extraction according to an orthodontic treatment plan. All teeth were collected with informed and oral consent from the patients and the parents according to ethical guidelines set by the French law (Loi Bioéthique n°2004–800) and with a special authorization for our team (n°DC-2009–927, Cellule Bioéthique DGRI/A5, direction générale pour la recherche et l’innovation, Ministère de l’enseignement supérieur et de la recherche, Paris, France).

### Synthetic ASARM Peptides

Phosphorylated ASARM (p-ASARM with 3 phosphoserine residues) and nonphosphorylated ASARM (np-ASARM) peptides were synthesized according to the human MEPE-derived sequence as previously reported [14], and were RDDSSESSDSGS(PO_3_H_2_)SS(PO_3_H_2_)ES(PO_3_H_2_)DGD and RDDSSESSDSGSSSESDGD, respectively.

### Cell Culture

Culture of pulp stem cells from human exfoliated deciduous teeth (SHEDs) were established as previously reported [40]. Briefly, after decontamination with povidone-iodine solution (Betadine, Meda Pharma, France), teeth were sectioned longitudinally and exposed pulp tissues were collected and enzymatically digested with type I collagenase (3 mg/ml; Worthington Biochem, Freehold, NJ, USA) and dispase (4 mg/ml; Boehringer Mannheim, Germany). Single-cell suspensions were obtained by passing the digested tissues through a 70 µm cell strainer. Cells were then seeded at a density of 10^4^/cm^2^, and the cultures were maintained with Dulbecco’s Modified Eagle Medium 1****g/L D-Glucose (DMEM; Invitrogen, Grand island, NY, USA) supplemented with 10% fetal bovine serum (FBS; Invitrogen), 1% penicillin/streptomycin (PS; Invitrogen), at 37°C with 5% CO_2_. The medium was refreshed the next day after initial cell attachment and then after at 3 times per week. Cells were detached by trypsinization at 70–80% confluence (0.25% trypsin EDTA solution Sigma-Aldrich, St. Louis, MO, USA) and either re-plated at the same density or frozen at −80°C. For all experiments, SHEDs were used between passages 2 and 4. Their SHED phenotype (CD90+, CD29+, CD44+, CD45−, CD73+, CD105+, CD146+, and 10% of STRO-1+) was confirmed by polychromatic flow cytometry analysis (LSRII, Becton Dickinson, NJ; antibodies from Biolegend, CA, BDBiosciences, NJ and eBioscience, CA) (Fig. S1).

### Tooth Slice Preparation

Caries-free human third molars extracted for orthodontic treatment purposes were collected from healthy young adults (18–25 years of age) with informed and oral consent of the patient, according to ethical guidelines set by the French law (see above). One-mm-thick tooth slices were prepared as previously described [41]. After disinfection with 70% ethanol, teeth were transversely sectioned at the cervical region using a diamond saw blade under cooling with sterile phosphate-buffered saline (PBS, Invitrogen) to obtain 1-mm-thick dentin slices. The pulp tissue was completely removed and tooth slices were immersed in 0.05% sodium hypochlorite for 10 s, then washed with sterile 1× PBS.

### 3D Culture Model Seeded with SHEDs

Tooth slices (one slice per well) were placed into wells of a 24-well culture plate for the cell culture studies. To provide a 3-dimensional (3D) cell culture environment, the empty pulp chamber was filled with 1 ml of polymerizing collagen gel prepared by mixing 10% (v/v) of ice-cold 10× DMEM (Sigma-Aldrich), 10% (v/v) of NaHCO_3_ (37 g/l), 50% (v/v) of type I collagen solution (2 mg/ml) and 30% (v/v) of SHEDs in suspension at 10^6^ cells/ml. To avoid adhesion and migration of the cells from the scaffold to the bottom of a conventional cell culture plate, cell culture-suspension plates were used (Cellstar, Greiner bio-one, Basel, Switzerland). The type I collagen used was extracted from rat tail tendons as previously described [42]. A sterile, stainless steel grid (Weber Metaux, Paris, France) was placed along the edge of the well to prevent gel contraction.

### Induction of the Odontoblast Differentiation Program

**Figure 1:**
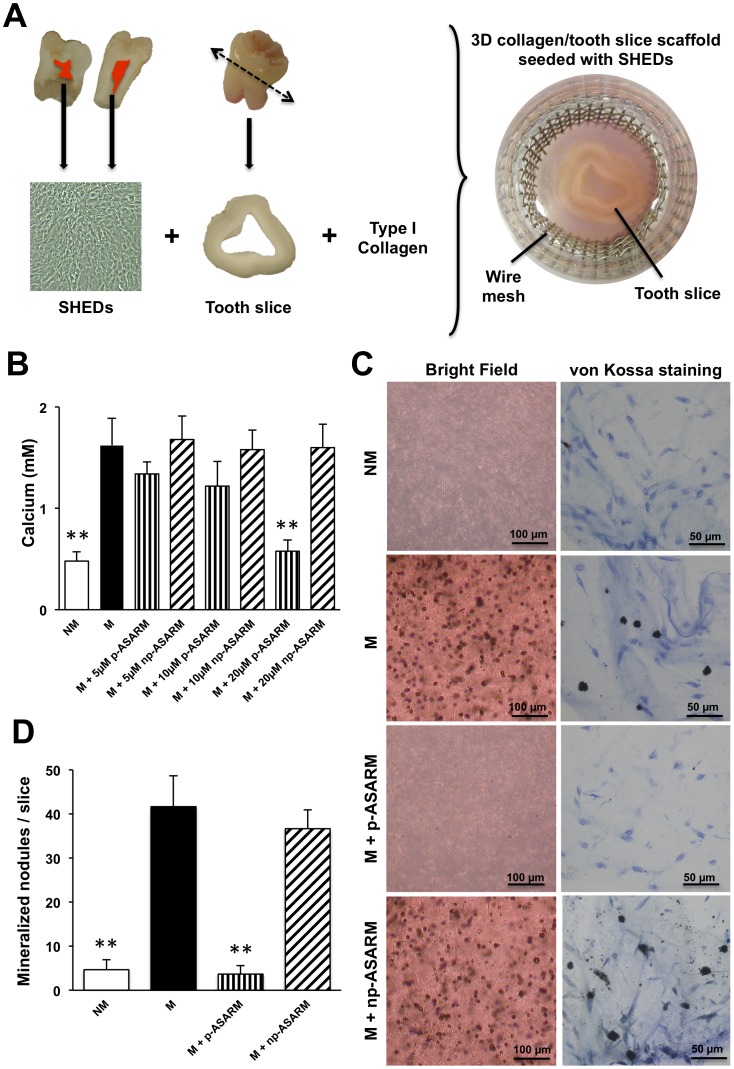
Inhibition of mineralization by phosphorylated MEPE-ASARM peptide in a 3D collagen/tooth slice culture model. A: Schematic of the 3D culture model. Stem cells from human deciduous exfoliated teeth (SHEDs) were obtained for cell cultures studies from the pulp of caries-free human third molars. Passaged cells (10^6^ SHEDs) seeded into a type I collagen gel scaffold were applied to a human tooth slice with a pulp chamber cavity to mimick the tooth/dentin environment, all of which were supported peripherally by a steel wire mesh to minimize collagen gel contraction. **B:** Calcium content in the cell/matrix layer determined by flame atomic absorption spectrometry of cultures maintained in NM or M conditions in the absence or presence of p-ASARM or np-ASARM at a concentration of 5, 10 or 20 µM for 21 days. Calcium content significantly decreased in the presence of 20 µM of p-ASARM while the nonphosphorylated form of the peptide had no effect on calcium content of the cultures. **C-D:** SHED cultures were maintained in nonmineralizing (NM) or mineralizing (M) conditions (see Materials and Methods) in the absence or presence of 20 µM phosphorylated (p-ASARM) or nonphosphorylated (np-ASARM) peptide for 21 days. Mineralized nodules in the extracellular matrix were clearly visible by light microscopy after von Kossa staining in the M and M+np-ASARM. Quantification of mineralized nodules (**D**) shows that they were essentially undetectable in presence of the p-ASARM peptide, whereas about 40 nodules were counted per slice in the M and M+np-ASARM samples. *n* = 3, error bars +/− SD, ** indicates significant difference (p<0.01) relative to mineralizing condition without peptide.

To induce the odontoblast differentiation program in the SHEDs, cells seeded into the 3D collagen/tooth slice scaffold (Fig. 1A) were cultured up to 21 days at 37°C with 5% CO_2_, 10% FBS, and 1% penicillin/streptomycin in supplemented DMEM in the presence of 10 mM ß-glycerophosphate (a source of phosphate for mineralization), 50 µg/ml ascorbic acid (promotes collagen secretion and matrix assembly), and of 10^8 ^M dexamethasone (which induces expression of biomineralization markers) − this is termed the mineralization condition (M) in our study [43]. Using this mineralization condition, either p-ASARM or np-ASARM (at concentrations of 5, 10, 20 µM for both) were added to the cultures during media changes 3 times per week. The negative control, termed the nonmineralizing condition (NM), consisted of DMEM cultures supplemented with 10% FBS and 1% PS. In all cases, medium was changed 3 times a week. Samples were collected at days 7, 14 and 21. At each time point and for each condition, cell morphology was observed using an inverted microscope (Olympus CKX41, Tokyo, Japan). All experiments were repeated in triplicate with cells from the same donor.

### Mineralization Assays

For assessing mineralized nodule formation after 3 weeks of culture, SHED-seeded 3D collagen/tooth slice scaffolds were fixed overnight at 4°C in 4% paraformaldehyde (PFA) solution and embedded in methyl methacrylate (Merck, Darmstadt, Germany) without any demineralization process [44]. Nine-micrometer-thick tissue sections were prepared, and mineralization in the ECM was revealed by von Kossa staining carried out by applying a 5% silver nitrate solution (Sigma) to the sections. Sections were then counterstained with toluidine blue. Mineralized nodules were counted in 30 randomly analyzed fields for each condition at 20× magnification and results are expressed as the number of nodules/tooth slice scaffold.

Quantification of calcium content extracted from mineral deposits within the cell/matrix layer after 21 days of culture was also assessed. For this, the 3D collagen scaffolds seeded with the SHEDs were gently removed from the tooth slice, rinsed twice with PBS, and decalcified with 0.5 M HCl for 1 hr at room temperature under constant agitation. After centrifugation at 2.150 g for 10 min, the calcium content in the supernatant was determined by the flame atomic absorption spectrometry method (AAnalyst, Perkin Elmer, Waltham, MA, USA).

### Immunohistochemistry

After 3 weeks of culture, SHED-seeded 3D collagen/slice scaffolds were fixed overnight at 4°C in 4% paraformaldehyde (PFA) solution, and embedded in methyl methacrylate (Merck, Darmstadt, Germany) without any demineralization process [44]. Nine-micrometre-thick tissue sections were prepared and endogenous peroxidases were blocked by incubation of the sections with 5 mM periodic acid at room temperature for 10 min. After extended rinsing in PBS, background activity was blocked at room temperature for 90 min using 1% bovine serum albumin (BSA) in PBS. Mouse monoclonal antibodies for osteocalcin (ab13420, Abcam, Cambridge, UK) and DSP (dentin sialoprotein, LFMb-21, Santa Cruz Biotechnology, CA, USA), and rabbit polyclonal antibodies for the MEPE mid-region and PHEX [14] were used all at a 1/200 dilution. Sections were treated in a moist chamber overnight at 4°C and further incubated for 90 min with a polyclonal goat anti-mouse immunoglobulin/HRP conjugate (Dako, Glostrup, Denmark) or with a polyclonal swine anti-rabbit immunoglobulin/HRP conjugate (Dako). Peroxidase visualization was obtained over 45 s in a dark chamber using 3.3′-diaminobenzidine tetrahydrochloride (Fast Dab, Sigma-Aldrich). The sections were counterstained with hematoxylin staining. Controls were carried out by omitting the primary antibody to exclude nonspecific binding. For PHEX, a goat anti-rabbit immunoglobulin-G Alexa Fluor 488 was used at a dilution of 1/200 (Sigma-Aldrich) in a dark-chamber, and nuclei were counterstained with dapi (Invitrogen). Microscopic images were merged using Image J software ver1.45p (public domain image processing and analysis software developed at the National Institutes of Health, Bethesda, MD, USA).

### Scanning Electron Microscopy

After 3 weeks of culture, 3D collagen/tooth slice scaffolds were fixed overnight in 1% PBS-buffered glutaraldehyde at 4°C and processed for scanning electron microscopy (SEM). Samples were fractured in liquid nitrogen and carbon- or gold-sputtered surfaces were observed in a scanning electron microscope (Cambridge S260 SEM, UK) equipped with an Everhart-Tornley and back-scattered electron detector (BSE) and with an energy-dispersive X-ray spectrometer (EDX) for X-ray microanalysis (Bruker Axs, WI, USA) with Esprit2 software.

### Light Microscopy and Transmission Electron Microscopy

Light microscopy and transmission electron microscopy (TEM) were used to analyze cells and mineralization of the 3D collagen/tooth slice scaffolds. Scaffolds were fixed overnight at 4°C in 4% paraformaldehyde and embedded in methyl methacrylate (Merck, Darmstadt, Germany). One-µm-thick survey sections were cut for light microscopy using a Reichert Ultracut S microtome, and sections were stained with von Kossa reagent (5% silver nitrate solution, Sigma) to reveal mineralization sites within the scaffold, and then counterstained with toluidine blue. Thin sections (90 nm) were cut and placed onto an electron microscopy grid followed by conventional staining with uranyl acetate and lead citrate at room temperature, and some sections were additionally subsequently stained with von Kossa reagent (5% silver nitrate solution, Sigma). Mineralized regions of the scaffolds were examined, and images recorded, using a Leitz DMRBE optical microscope equipped with a Sony DXC-950 CCD camera, and using a JEOL 1011 TEM operating at 80 kV and equipped with a GATAN Erlangshen camera 1000.

### RT-PCR Analysis for Gene Expression

The mineralizing potential of the SHED cultures treated with the ASARM peptides was investigated by analyzing the expression of type I collagen α-chain (*COL1*), dentin sialophosphoprotein (*DSPP*), *MEPE*, *PHEX*, osteocalcin, and tissue-nonspecific alkaline phosphatase (*ALPL*) genes after extraction of total RNA from the cultures after 7, 14 and 21 days using RNeasy Mini kit (Qiagen, Hilden, Germany). 300 ng of total RNA was reverse-transcribed with Superscript II (Gibco, Invitrogen) using oligodT. cDNA was amplified with specific primers (Eurofins mwg operon, Ebersberg, Germany) in a SYBR® Green PCR Master Mix (Roche, Basel, Switzerland). Each primer pair was chosen for best efficiency and controlled for specificity (Table 1). Quantitative real-time PCR (qPCR) analysis was carried out using LigthCycler 480 [45]. The PCR cycles consisted of an initial pre-incubation step (95°C for 5 min), followed by 45 cycles of amplification (95°C for 5 s, 55°C for 5 s, 70°C for 10 s). Relative gene expression levels were estimated using the deltaCq method [46]. *GAPDH* and *ubiquitine C* (*UBC)* were determined using GeNorm software on a set of 5 standard housekeeping genes and were used as housekeeping genes for the rest of the study. Results were expressed as mean ± SD of relative mRNA level for each time point and for the different conditions.

**Table 1:** Primer design for RT-PCR analysis.

	5′ –3′ Forward primer	5′ –3′ Reverse primer	Product
*MEPE*	GAGTTTTCTGTGTGGGACTACT	GCTTTGCTTAGTTTTCTCAGTC	86-bp
NM_020203.2			
*DSPP*	GAGGAGATGCTTCTTATAACTCTG	GTGCTATCACTGTCATCATCTT	91-bp
NM_014208.3			
Osteocalcin	GCACCCTTCTTTCCTCTT	GGAGTTTATTTGGGAGCAG	95-bp
NM_199173.3			
*COL1*	TGACTGGAAGAGTGGAGAGTA	TCTTGCTGATGTACCAGTTCT	149-bp
NM_000088.3			
*ALPL*	AACTGATGTGGAGTATGAGAGTG	GAAGTGGGAGTGCTTGTATCT	112-bp
NM_000478.4			

### Total Protein Extraction and Western Blot Analysis

At 7, 14 and 21 days, the collagen matrices seeded with SHEDs were gently removed from the tooth slices and processed for total protein extraction using ice-cold extraction buffer (each collagen scaffold was placed in 100 µl buffer: 50 mM Tris-HCl, pH 7.5, containing 5 mM EDTA, 150 mM NaCl and 0.2% Triton X-100), supplemented with 1/100 Protease Inhibitor Cocktail Set V EDTA free (Calbiochem, La Jolla, CA, USA). The resulting homogenates were briefly sonicated on ice, cleared by centrifugation at 10,000 g for 10 min at 4°C and stored at −80°C. Homogenates (10 µg) were subjected to 10% SDS-PAGE and transferred onto a nitrocellulose membrane (Bio-Rad). Membranes were incubated overnight at 4°C with monoclonal anti-DSP (1/2000; LFMB-21, Santa Cruz) and anti-osteocalcin (1/1000; ab13420, Abcam) antibodies, or polyclonal anti-MEPE mid-region and anti-PHEX antibodies at 1/2000 dilution [14]. Membranes were incubated with a peroxidase-linked swine anti-rabbit secondary antibody (1/10,000) for 2 hrs at room temperature, then rinsed and followed by enhanced chemiluminescence detection of bound primary antibodies (Roche Diagnostics, Meylan, France). As a control for protein loading, membranes were carefully washed in stripping buffer (Pierce Chemical, Rockford, IL, USA) and processed with a monoclonal mouse anti-α tubulin antibody (clone B-5-1-2, Sigma). Quantification was performed using Image J software.

### Implantation of ASARM Peptides in a Rat Pulp/Dentin Injury Model

Affi-Gel agarose beds (Bio-Rad Laboratories) were soaked in a solution of p-ASARM or np-ASARM dissolved in PBS (2 µg/µl) for 2 hrs, and dried overnight at 37°C. Beads soaked with PBS were used as a control. The beads were implanted into the pulp of the upper first molar of 9 rats (6 week-old, OFA/SD Charles River, Lyon, France) using a surgical procedure previously described [47]; this study was designed according to the ARRIVE guidelines and approved by the Animal Care Committee of the University Paris Descartes (agreement CEEA34.CC.016.11, Comité d’éthique pour l’expérimentation animale n° 34, Paris, France). Each animal received two of the three compared treatments on the left and right first maxillary molars. Treatments were then analyzed between animals using the molar as the statistical unit. Briefly, after gingival electrosurgery, 18 half-moon-shaped class V 0.5 mm cavities were prepared on the mesial aspect of the first upper molar using a 0.2-mm-diameter round bur (E0123, Dentsply Maillefer, Ballaigues, Switzerland). Pulp exposure was accomplished with a root-canal-shaping rotary nickel-titanium file system (Protaper, Dentsply), after which 10 peptide-treated beads − either with buffer (n = 6), or p-ASARM (n = 6), or np-ASARM (n = 6) − were implanted into the pulp using a blunt steel probe. After bead placement, cavities were filled with Biodentine cement (Septodont, Saint-Maur des Fossés, France). The rats were sacrificed after one month and X-ray microcomputed tomography (micro-CT) analysis was performed on the treated molars (Skyscan Model 1172, Kontich, Belgium). Semi-automatic segmentation of X-ray serial images allows the calculation of the unmineralized pulp volume in order to indirectly quantify the mineralization of the reparative dentin within the teeth. After X-ray scanning, tissue demineralization was then performed in 4.13% EDTA for approximately 8 weeks. After tissue embedding in paraffin (Paraplast), 7-µm-thick sections were cut on a rotary microtome and either prepared for staining with hematoxylin-eosin stain, or prepared for immunohistochemistry. Sections were deparaffinized and rehydrated before blocking with 10% normal goat serum for 45 min at room temperature. Sections were incubated overnight at 4°C with primary antibody against the MEPE mid-region at a 1/200 dilution and processed as described above in the immunohistochemistry section.

### Human XLH Tooth Characterization

Patient characteristics are given in Table 2. All teeth were obtained with the informed and oral consent of the patients and the parents, according to ethical guidelines set by the French government (see above). Human deciduous incisors were collected from a 3-year-old male patient with XLH [26]. Immediately after extraction, teeth were fixed for 7 days at 4°C in a 4% paraformaldehyde solution buffered at pH 7.3 by PBS and processed after demineralization [7] for Sirius red staining.

**Table 2:** Clinical and biological characteristics of the patients with XLH at the time of tooth extraction.

Patients with XLH	1	2
Sex	M	F
Age (yr)	3	15
*(at the time of tooth collection*)		
		
*Treatment*	–	+
Age at onset (yr)	*not yet started at the time*	3.3
Duration (yr)	*of tooth collection*	10
Unalfa® (µg/day)	–	1.25
Phosphates (mg/day)	–	2100
Compliancy	–	moderate
		
*Signs of rickets*		
Leg bowing	+++	+
Height (SD)	−3	−2.1
Serum P (mmol/l)	0.63	0.58
Alkaline phosphatase (IU/L)*	498	523
		
*Dental status*	vital	vital
DMFT or dft**	7	2
Collected tooth	61,71	38, 48
Type	deciduous	permanent
Pulp status	vital	vital
Reason of extraction	(trauma)	(orthodontic treatment
		plan)

* (normal range is inferior to 300 IU/L).

** DMFT: Decayed Missing Filled permanent Teeth; dft: decayed filled deciduous teeth.

Human permanent third molar germs were collected in the context of an orthodontic treatment plan from a 15-year-old female patient with XLH [26]. Immediately after extraction, teeth were fixed for 7 days at 4°C in 70% ethanol, included in methyl methacrylate [44] and processed for von Kossa and Sirius red staining.

### Statistical Analysis

Values are represented as the mean +/− standard deviation (SD). Data were analyzed by one-way analysis of variance (ANOVA) followed by post hoc Fisher’s least significant difference test.

## Results

### Dose-dependent Response of SHEDs to p-ASARM Peptide

SHEDs were cultured in a 3D collagen matrix enclosed within a tooth slice under mineralizing conditions (Fig. 1A). Cells were treated at concentrations ranging from 5 to 20 µM of phosphorylated MEPE-derived ASARM peptide (p-ASARM) for 21 days in mineralizing medium (Fig. 1B). p-ASARM peptide was shown to inhibit mineralization nodule formation in a dose-dependent manner with the highest inhibition occurring at the highest concentration (20 µM) of p-ASARM, which is in agreement with preliminary data obtained with 2D cultures of either human dental pulp stem cells or SHEDs (data not shown), and with previous studies [33], [38]. This 20 µM concentration was subsequently used for all other experiments. In contrast, nonphosphorylated MEPE-derived ASARM peptide (np-ASARM) had no effect on mineralization as previously reported with other cell types [14], [33].

### p-ASARM Peptide Inhibits ECM Mineralization in a 3D Culture of SHEDs

To examine the role of p-ASARM on ECM mineralization, SHEDs were cultured for 21 days in the 3D model under mineralizing (M) or nonmineralizing (NM) conditions, or under mineralizing conditions with either 20 µM of p-ASARM (M+p-ASARM) or np-ASARM (M+np-ASARM). No mineralization was observed in the presence of p-ASARM, whereas readily distinguishable mineralized nodules were observed in the absence or the presence of np-ASARM (Figs. 1C). Calcium quantification from the cell cultures performed by flame atomic adsorption spectrometry confirmed the mineralized nodule counts by showing very low calcium levels in the M+p-ASARM treatment condition similar to the nonmineralizing condition calcium content (Fig. 1D).

**Figure 2:**
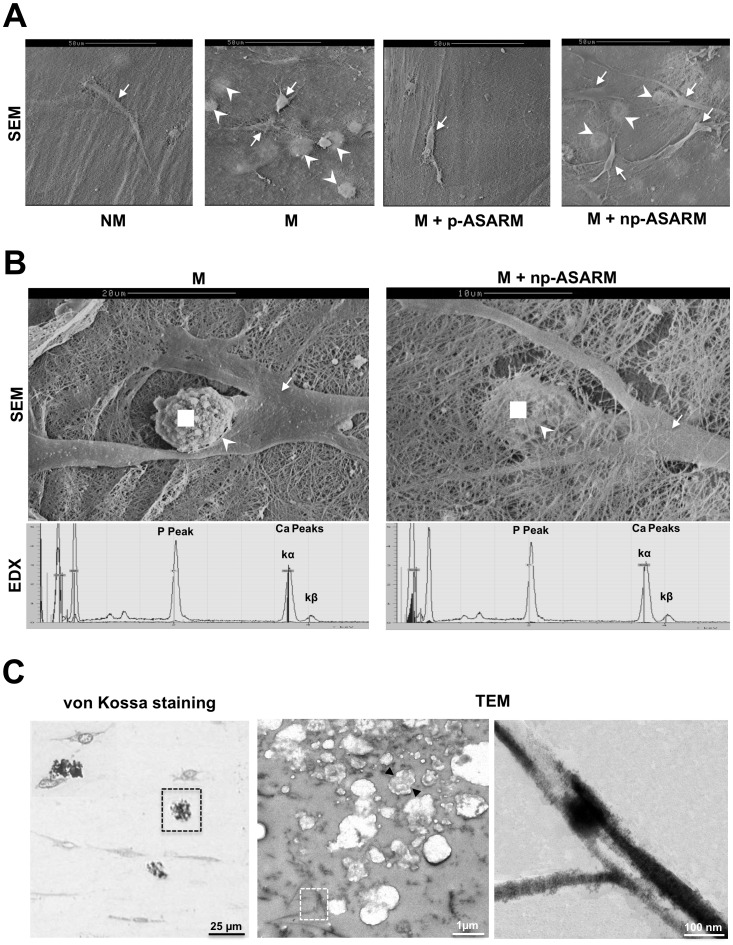
Light and electron microscopy of the cells, matrix and mineral in 3D SHED cell cultures. SHED cell cultures maintained in nonmineralizing (NM) or mineralizing (M) conditions in the absence or presence of 20 µM of either phosphorylated (p-ASARM) or nonphosphorylated (np-ASARM) ASARM peptide for 21 days were visualized by light microscopy and by scanning (SEM) and transmission (TEM) electron microscopy. **A,B:** SEM reveals SHEDs (arrows) well integrated into the collagen scaffold. Mineralization of the cultures appears as nodules within the collagen scaffold (arrowheads) only in the M and the M+np-ASARM conditions. Energy-dispersive X-ray spectroscopy (EDX) for compositional microanalysis of the nodules (performed at the white square) shows major spectral peaks for calcium (Ca) and phosphorus (P) with an acquired Ca/P ratio of 1.67+/−0.05 in both mineralizing conditions where nodules appeared. **C:** Light microscopy (left panel) and TEM (center and right panel) of the mineralized cultures (M and M+np-ASARM). Mineralized nodules (black box, left panel) are often in close proximity to the SHED cells, and consist of aggregates of multiple, smaller mineralization nodules (arrowheads) and occasional mineralized collagen fibrils (white box center panel, and right panel).

Analysis of samples by scanning electron microscopy (SEM) of fractured collagen/tooth slice scaffolds showed mineralized nodules within the collagenous ECM in close association with neighboring cells (Figs. 2A, B). In all conditions, well-distinguishable cell processes were observed in contact with the tooth slice (Fig. S2). Calcium-to-phosphate ratios of mineralized nodules obtained by EDX spectroscopy were close to 1.67 for the two conditions permissive of mineralization (M, and M+np-ASARM, Fig. 2B), which is consistent with the formation of a hydroxyapatite-like mineral phase. In contrast, cell cultures treated under NM conditions, or with the M+p-ASARM condition, showed no evidence of mineralization (Fig. 2A). Under the conditions where mineral formed, analysis by light microscopy and by TEM of mineralized scaffold matrix regions showed a small nodular type of mineralization amongst the collagen fibrils, with some mineralization extending occasionally along collagen fibrils (Fig. 2C); no such mineralization was observed in cultures treated with p-ASARM (data not shown).

### p-ASARM does not Modify Type I Collagen mRNA Expression but Upregulates Tissue-nonspecific Alkaline Phosphatase mRNA

**Figure 3:**
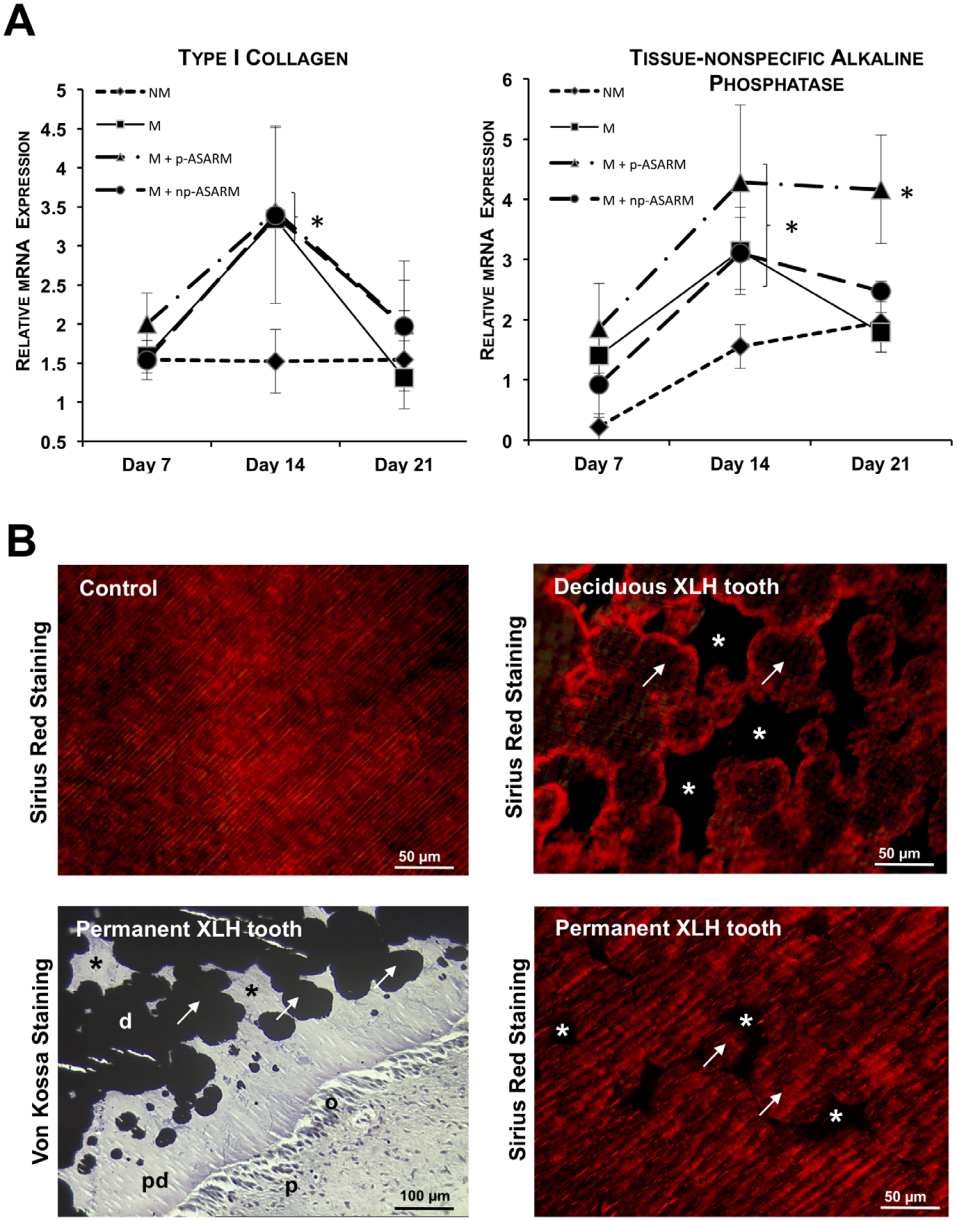
Effect of MEPE-ASARM peptides on type I collagen and tissue-nonspecific alkaline phosphatase expression. A: Quantitative real-time PCR (as described in Materials and Methods) for type I collagen and tissue-nonspecific alkaline phosphatase expression in 3D cultures of SHEDs maintained in nonmineralizing (NM) or mineralizing (M) conditions in the absence or presence of either 20 µM phosphorylated (p-ASARM) or nonphosphorylated (np-ASARM) ASARM peptide for 21 days. mRNA expression levels were upregulated from day 7 to day 14 in M, M+p-ASARM and M+np-ASARM conditions and, at day 21, returned to a level comparable to that of the NM condition, except for alkaline phosphatase which remained high in the M+p-ASARM condition. *n* = 3, error bars +/− SD, * indicates significant difference (p<0.05) relative to NM condition. **B:** Sirius Red staining of collagen in deciduous tooth sections from a 3-year-old male XLH patient (upper right panel), permanent tooth sections from a 15-year-old female XLH patient (bottom right panel) and control patient (upper left panel) revealing an intact collagen distribution only where dentin has mineralized in the form of characteristic calcospherites (arrows), and not in the interglobular spaces (asterisks) in the dentin where mineralization is typically impaired in XLH and where matrix degradation occurs. Sirius Red staining of normal dentin (right panel) is generally homogeneous across the tubular dentin. Similarly, von Kossa staining counterstained with toluidine blue in the same XLH permanent tooth sections (bottom left panel) show lack of fusion of calcospherites (arrows) with large non-mineralized interglobular spaces (asterisks) in the dentin. o: odontoblast. pd: predentin. d: dentin. p: pulp.

The aim of seeding SHEDs in a 3D collagen matrix was to generate an ECM environment mimicking that found in vivo which favors the cell differentiation process of progenitor/stem cells [48], [49], [50]. We therefore investigated some of the major ECM components and marker proteins known to be expressed by SHEDs as they begin to differentiate towards the odontoblast phenotype. The number of transcripts encoding type I collagen − the major ECM protein of odontoblasts/dentin − increased under mineralizing conditions from day 7 to day 14 (approximately 2-fold), after which it returned at day 21 to the basal expression level of cells grown under the nonmineralizing condition (Fig. 3A); this induction was not modified by the presence of the ASARM peptides. Likewise, alkaline phosphatase mRNA expression was upregulated in all mineralizing conditions between days 7 and 14 (Fig. 3A). Thereafter, it remained stable up to day 21 in the presence of p-ASARM, but diminished to a level comparable to the nonmineralizing condition in the other conditions (M and M+np-ASARM).

As collagen has never been investigated in teeth from patients with XLH, we examined its distribution in tooth sections. Sirius red staining, which stains collagen fibrils, was associated predominantly with the calcospherites characteristic of this pathology, indicating that there was little or no collagen (degraded) in the interglobular spaces (Fig. 3B) as previously shown [12].

### Odontoblast Differentiation is Impaired in the Presence of ASARM Peptides

**Figure 4:**
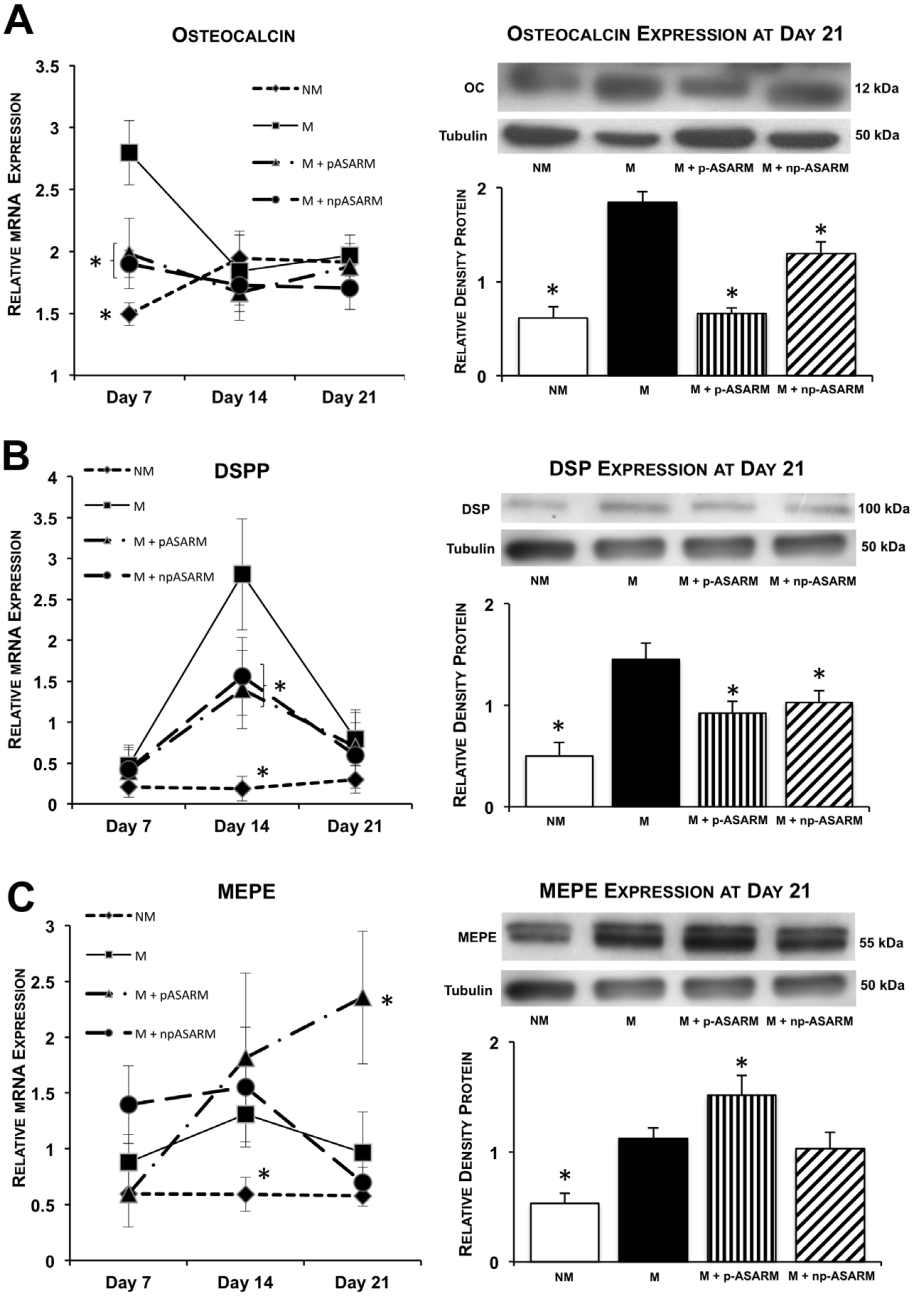
Effect of MEPE-ASARM peptides on osteocalcin, DSPP and MEPE expression. SHED cell cultures were maintained in nonmineralizing (NM) or mineralizing (M) conditions in the absence or presence of 20 µM of either phosphorylated (p-ASARM) or nonphosphorylated (np-ASARM) ASARM peptide for 21 days. Quantitative real-time PCR at day 7, 14 and 21, and Western blotting at day 21, for osteocalcin, DSPP/DSP and MEPE were performed. **A,B:** Osteocalcin and DSPP/DSP expression are induced under the M condition both at the mRNA and protein levels. This induction is reduced in the presence of both the p-ASARM and np-ASARM peptides. **C:** mRNA and Western blot analysis for MEPE reveal increased expression of MEPE in all mineralizing conditions from day 7 to day 14 (expression was not detectable at baseline). At day 21, MEPE expression was still strongly upregulated in the M+p-ASARM condition. *n* = 3, error bars +/− SD, * indicates significant difference (p<0.05) relative to the mineralizing condition without peptides (M).

**Figure 5:**
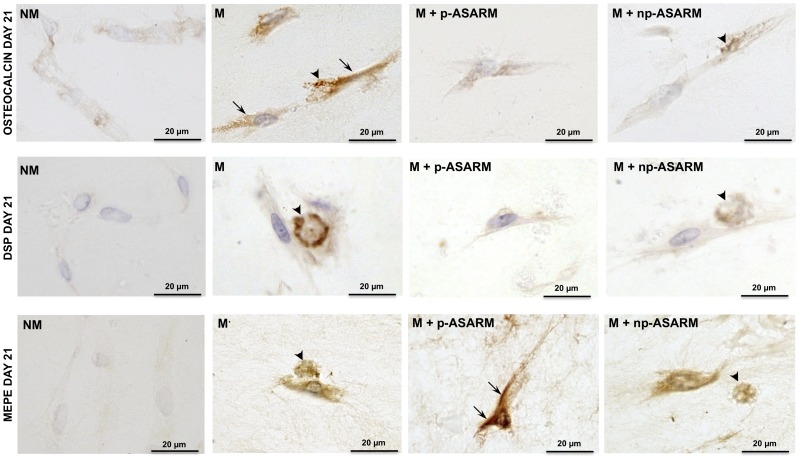
Immunohistochemical detection of osteocalcin, DSP and MEPE after treatment with MEPE-ASARM peptides. SHED cell cultures were maintained in nonmineralizing (NM) or mineralizing (M) conditions in the absence or presence of 20 µM of either phosphorylated (p-ASARM) or nonphosphorylated (np-ASARM) ASARM peptide for 21 days. Immunohistochemistry for osteocalcin, DSP and MEPE was performed on methyl methacrylate sections of 21-day cultures. Osteocalcin immunostaining (top row) is strong in SHEDs (arrows) and nodules (arrowheads) in the M and M+p-ASARM conditions. Immunohistochemistry for DSP shows strong staining in mineralized nodules in both the M and M+np-ASARM conditions. MEPE immunostaining is found in SHEDs and nodules in mineralizing conditions the (M and M+np-ASARM conditions) but is particularly strong in the cultures treated with p-ASARM that do not mineralize (M+p-ASARM). The images presented are representative of all sections examined.

Differentiation of SHED cells to odontoblasts is associated with an increase in mineralization-regulating protein marker gene expression. Osteocalcin mRNA was increased in all mineralizing conditions at day 7 relative to the nonmineralizing condition, an expression pattern substantially reduced in the presence of the ASARM peptides (Fig. 4A). Western blot analysis confirmed a lower expression of the osteocalcin protein in the presence of the ASARM peptides and most particularly after addition of p-ASARM. Osteocalcin immunolocalization revealed variable levels of cell immunostaining under all conditions. In addition, mineralized nodules in the mineralizing conditions (M and M+np-ASARM) were stained for osteocalcin (Fig. 5, top row, arrows) whereas no ECM labeling was observed in the p-ASARM-treated samples and in the nonmineralizing condition. Analysis of the expression of the dentin sialoprotein protein (DSP) precursor protein DSPP, another marker of odontogenic differentiation [51], revealed an increase in its transcript number at day 14 in all mineralizing conditions as compared to the nonmineralizing condition (Fig. 4B). However, in the presence of the ASARM peptides, this upregulation was twice as low when compared to control mineralizing conditions. Such a differential expression pattern was confirmed at the protein level by Western blot analysis at day 21. For DSP, positive immunohistochemical staining was revealed in the mineralized nodules in the ECM (Fig. 5, middle row, arrows in M and M+np-ASARM conditions).

### p-ASARM Peptide Upregulates MEPE Expression

Given the mineralization-inhibiting properties of the MEPE-derived p-ASARM peptide and also its effects on expression of odontoblast marker proteins, we next examined the expression of *MEPE* itself after the 3D cultures were treated with the ASARM peptides. As presented in Figure 4C, MEPE transcript expression was induced from day 7 to day 14 in all mineralizing conditions, regardless of the presence of the ASARM peptides. However, at day 21, MEPE transcripts remained significantly increased (2.8-fold) in the presence of p-ASARM peptide, whereas they returned to levels in the other mineralizing conditions (M and M+np-ASARM) comparable to those observed in nonmineralizing conditions. This result was confirmed by Western blot analysis where MEPE protein expression increased in all mineralizing conditions (Fig. 4C), but its expression was significantly higher in the presence of p-ASARM. Consistent with this, immunohistochemistry for MEPE showed particularly strong cell staining for the p-ASARM treatment under mineralizing conditions (Fig. 5, bottom row, arrowheads). In contrast, in the presence of np-ASARM or in the mineralizing condition alone without added peptide, the staining was associated with the mineralized nodules in the ECM, with moderate staining of the cells (Fig. 5, bottom row, arrows). These results indicate that the p-ASARM peptide increases MEPE expression by differentiating SHEDs via a positive feedback mechanism. Given that MEPE has protein-protein interactions with PHEX, and given that its ASARM peptide is a substrate for PHEX enzymatic activity, we examined PHEX expression *in vitro* under our different treatment conditions applied to the SHED cells in 3D cell culture. In this collagen/tooth slice scaffold model, PHEX expression was not altered by ASARM treatment (Fig. S3).

### p-ASARM Peptide Impairs Dentin Formation in a Rat Model of Pulp Repair

**Figure 6:**
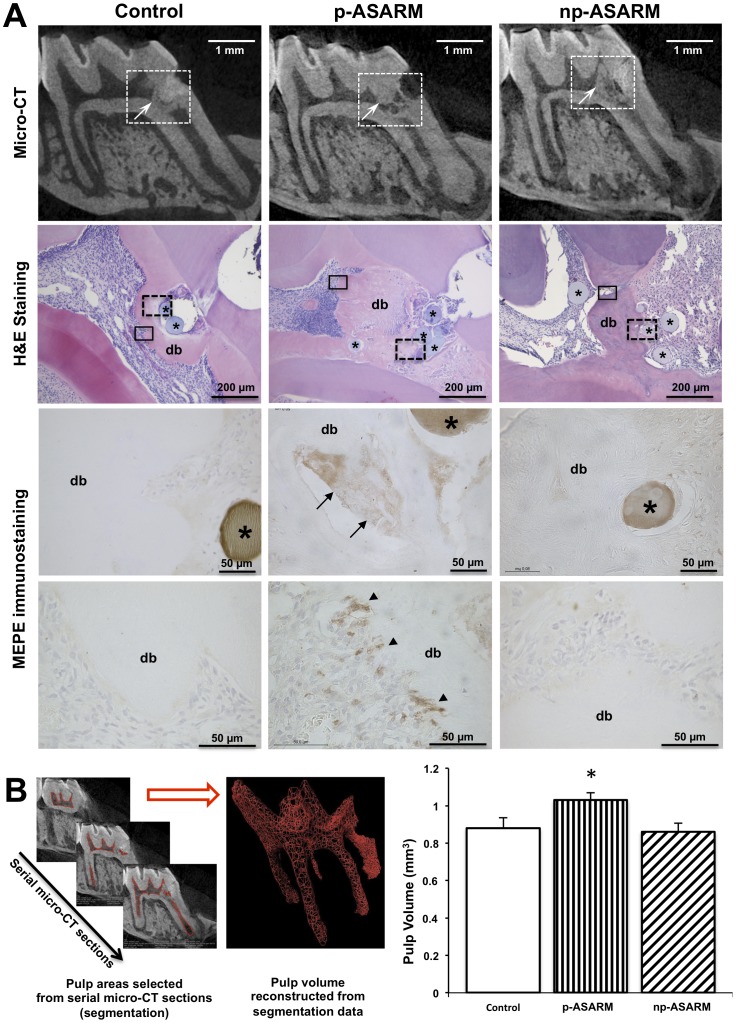
Effect of MEPE-ASARM peptides on reparative dentin mineralization and MEPE expression in a rat dentin/pulp wound model. This *in vivo* data is from rats sacrificed at 1 month after implantation of agarose beads soaked in either p-ASARM or np-ASARM, or buffer (control), into the pulp of the first upper molar. A: MEPE derived p-ASARM impairs reparative dentin mineralization and enhances MEPE expression. Micro-computed tomography (micro-CT) sections (top row) show mineralization (arrows) occurring in the pulp chamber as a reparative process (dentin bridge formation) near the pulp injury (white dashed box outline). The dentin bridge appears thinner and irregular with large voids (mineralization defects) in the p-ASARM treatment when compared to the control and np-ASARM treatment. Hematoxylin and eosin (H&E) staining of paraffin histology sections (second row) taken from the region outlined by the white dashed boxes show the irregular nature of the dentin bridge (db) in the vicinity of the beads (asterisks) soaked in p-ASARM as compared to the control and the np-ASARM treated wounds. Immunohistochemistry for MEPE (third row) in the region demarcated by the black dashed box outlines shows an accumulation of MEPE (arrows) in nonmineralized spaces of the reparative dentin bridge only in the case p-ASARM treatment. Immunohistochemistry for MEPE (bottom row) in the region demarcated by the black box outlines shows strong MEPE staining of cells (odontoblasts) secreting the dentin bridge. In control and np-ASARM-treated samples, the dentin bridge appears homogenous and with no or weak staining for MEPE. B: Quantification of the pulp volume (to indirectly measure mineralization volume “pulp fill”) was performed by micro-CT of the bead-implanted molars. Semi-automatic segmentation (red area) of the pulp was performed from micro-CT serial sections in order to calculate the global volume of the pulp. The volume of the pulp from the p-ASARM-treated molars was significantly higher when compared to the control and np-ASARM treatment suggesting that mineralization in the reparative dentin process was impaired by p-ASARM. * indicates significant difference (p<0.05) relative to control.

To evaluate whether ASARM peptides display similar properties on dentin mineralization in teeth *in vivo*, we implanted agarose beads soaked in either p-ASARM or np-ASARM peptide, or control buffer, into injured dental pulp in a tooth-wound model in young rats. In this model, the repair process is mediated by endogenous pulp cells undergoing odontoblast differentiation [47]. Micro-CT analysis and histology after hematoxylin and eosin staining at 1 month following surgery and peptide implantation showed a reparative mineralized dentin bridge in all samples (Fig. 6A); however, it appeared thicker in both control and np-ASARM treated pulps when compared to the p-ASARM treated teeth. To quantify this reparative process using micro-CT, the pulp volume was segmented in order to calculate its global volume (Fig. 6B). The pulp volume was significantly greater in the p-ASARM-treated samples (1.03****mm^3^+/−0.04) (thus less mineralized dentin bridge tissue) than in both the control and np-ASARM conditions (0.88****mm^3^+/−0.06 and 0.86****mm^3^+/−0.05, respectively). These results in an animal model are consistent with our other *in vitro* SHED cell culture observations and show that the p-ASARM peptide impairs dentin neo-formation and its mineralization after pulp injury. Given our *in vitro* data indicating that mineralization inhibited by p-ASARM was accompanied by an increased expression of MEPE, we then evaluated MEPE expression in this *in vivo* model using an antibody directed against the mid-region of MEPE. Nonmineralized spaces within a heterogeneous dentin bridge stained positively for MEPE in the p-ASARM treated samples (Fig. 6A). In addition, cells adjacent to and likely secreting this dentin bridge were strongly stained for MEPE. In contrast, in control and np-ASARM samples, a homogenous reparative bridge was seen with no MEPE staining. Taken together, these results demonstrate that the p-ASARM peptide enhances MEPE expression by differentiating dental pulp stem cells both *in vivo* and *in vitro*.

## Discussion

A MEPE-derived ASARM peptide was previously identified in human hypophosphatemic tooth dentin [12]. Together with the results of the present study, these data present evidence that this peptide (and potentially other similar ASARM peptides derived from other matrix SIBLING proteins) may be involved in impairing tooth dentin mineralization as observed in patients affected with XLH. Using a cell culture collagen/tooth slice 3D scaffold model, together with an *in vivo* wound healing animal model, the present study demonstrates that the MEPE-derived ASARM peptide inhibits odontogenic differentiation of deciduous dental pulp stem cells (SHEDs), enhances MEPE expression, and impairs dentin ECM mineralization.

Cell functions such as migration, adhesion and differentiation are routinely studied in 2D cultures. However, interpretations from these *in vitro* models are limited by the fact that they do not accurately recreate the appropriate, natural tissue architecture [48], [52], [53], [54], [55]. We have therefore developed and used a 3D collagen/tooth slice scaffold model where seeded cells can undergo differentiation in a more natural ECM environment [41], as demonstrated by the expression of several well-known odontoblast markers commonly used to identify this cell type (without the added tooth slice, no odontoblast marker expression was observed – data not shown). Based on our previous clinical and histologic findings [3], [6] together with observations made by others [4], [8] reporting severe abnormalities in deciduous teeth of patients with XLH (spontaneous dental abscesses associated with enamel cracks, poorly mineralized dentin, large pulp chamber and prominent pulp horns), we used 3D culture of dental pulp cells which displayed a typical SHED phenotype [56] as confirmed by polychromatic flow cytometry (Fig. S1), and a tooth/dentin wounding model in rats to examine further the causes of XLH tooth pathology, with a focus on the effects of the MEPE ASARM peptide. Under mineralizing culture conditions without added peptide, we observed von Kossa-stained mineralized nodules in the ECM which were in close proximity to the cells and not more distal in the collagen scaffold, suggesting that they resulted from a cell-mediated biomineralization process rather than from random (dystrophic) mineral deposition within the collagen scaffold. Collagen mineralization along collagen fibrils was confirmed morphologically by SEM and TEM analyses and by microanalytical EDX demonstrating high calcium and phosphorus levels within the nodules with calcium/phosphate ratios being comparable to those observed in human mineralized bone and tooth extracellular matrices [57,58,59. In addition, cells differentiated from normal SHEDs expressed a variety of mineralization-relevant biomarkers including PHEX, which demonstrates the utility of our collagen scaffold *in vitro* model in providing a suitable 3D matrix environment enabling odontoblast cell differentiation. Taken together, these observations confirm that the culture model used here was appropriate to evaluate SHED cell differentiation in response to addition of p-ASARM – a circumstance thought to be relevant to bone and tooth mineralization in the pathologic XLH condition where available evidence describes an accumulation of ASARM-containing (and other) peptides derived from the SIBLING proteins [14], [32], [33], [36], [37], [60]. Whereas most of the studies reported to date describe the effects of ASARM peptides and their degradation by PHEX on mineralization in bone cell cultures, the present study is the first to extend these results to mineralization in odontoblast cultures, and to describe effects of ASARM on cell differentiation.

In terms of its effects on mineralization in the SHED/odontoblast 3D cultures, the phosphorylated form of the MEPE-derived ASARM peptide (p-ASARM) inhibits the formation of mineralization nodules in the collagenous ECM, whereas the non-phosphorylated form has no effect on mineralization. This observation is consistent with previous findings from bone cell cultures showing that the phosphorylation of MEPE- and OPN-derived ASARM peptides is necessary for the inhibition of ECM mineralization [14], [32], [33], [37], [60]. These previous bone cell studies demonstrated that the ASARM peptides inhibit mineralization in a phosphorylation-dependent manner via direct binding to hydroxyapatite crystals [14], [33]. Interestingly, the p-ASARM dosage used by us and others to inhibit mineralization in cell culture (20 µM) is similar to the mean levels of ASARM peptide directly measured in the serum of patients with XLH, and in the serum of homolog Hyp mice (16 and 23 µM, respectively) [61].

In terms of its effects on cell differentiation in the SHED 3D cultures, we have made here the additional finding that ASARM peptide impairs dental pulp stem cell differentiation into odontoblast-like cells, an important observation with direct relevance to the pathologic mechanisms underlying XLH. The modified expression of odontoblast markers (DSPP, osteocalcin, alkaline phosphatase and MEPE) in the presence of ASARM peptides is a novel finding for tooth cells, and we further show that such an influence is more pronounced after exposure to the phosphorylated form of the peptide. Such observations are consistent with a previous study conducted on murine bone marrow stromal cells differentiating into osteoblasts, which reports a modification of osteoblast gene expression in the presence of MEPE-derived ASARM peptide [14]. Contrary to this, another study reported no effect of ASARM on cell differentiation using the murine MC3T3-E1 pre-osteoblast cell line [33]. This discrepancy may be explained by differences in cell characteristics (*e.g.* stem cells versus partially committed cells) and by the fact that these previous studies were carried out in 2D cell cultures.

In the present study, odontoblast differentiation was substantially impaired in the presence of p-ASARM as evidenced by the decrease (both at the mRNA and protein levels) of markers related to the odontoblast program such as DSPP and osteocalcin, whereas there was only a slight effect on these markers when np-ASARM was added.

The decreased osteocalcin expression reported here is consistent with those previously reported in bone cell cultures treated with p-ASARM [14], and also with our report on dentin matrix proteins in XLH deciduous human teeth where we showed by Western blot analysis of dentin extracts a decrease in osteocalcin and DSP compared to age-matched controls [7]. In contrast, type I collagen, the most prominent protein of dentin ECM, was not modified by the addition of the ASARM peptides in our present cell culture study. Indeed, type I collagen expression followed a pattern comparable to that previously reported for osteogenic cells cultured in a collagenous ECM-like 3D scaffold and treated with ascorbic acid [53]. In hypomineralized XLH human dentin, it appears that ECM matrix components are degraded at sites where the dentin does not mineralize [7].

Expression of tissue-nonspecific alkaline phosphatase (ALPL, TNAP) was up-regulated in our experiments in all mineralizing conditions from day 7 to day 14. This phosphatase catalyzes the hydrolysis of most phosphomonoesters – particularly cleavage of the potent mineralization-inhibiting small molecule pyrophosphate (PPi) found in serum, most tissue fluids and ECMs – releasing inorganic phosphate (Pi) which could contribute to local phosphate levels and mineralization [62]. In the present study, mRNA levels of alkaline phosphatase were upregulated prior to ECM mineralization and downregulated when mineralized nodules became apparent by bright field light microscopy after von Kossa staining. This expression pattern has been previously reported during the differentiation of human and murine osteogenic cell lines cultured within a dense collagen scaffold [53], [63]. We additionally found that in the presence of the p-ASARM peptide, alkaline phosphatase mRNA levels remained elevated at the later culture time point (day 21). Such a difference might be explained by the fact that although most of the required conditions for mineralization *in vitro* have been met and are present in the cultures – including high levels of phosphate and calcium for mineralization, ascorbic acid for synthesis and secretion and assembly of collagen fibrils, and noncollagenous protein secretion – mineralization has not occurred in the presence of p-ASARM, and the cells are sensing that excess inhibitory PPi may need to be removed by continuing their ALPL expression. Interestingly, and similar to this observation, the serum of patients with XLH shows increased alkaline phosphatase activity, being especially high in children prior to their treatment (with vitamin D analogues and phosphate supplements) [64]. For example, in a group of 28 children affected with XLH and having a PHEX mutation, with a mean age of 2.2±1.1 years, the mean ± SE alkaline phosphatase activity before any treatment was 1,320±910****IU/L, being more than 4 times higher than the upper normal range (normal range is inferior to 300****IU/L); data from Centre de Référence des Maladies Rares du Métabolisme du Phosphore et du Calcium, AP-HP, Kremlin Bicêtre, France).

Our *in vivo* observations using a rat pulp injury model also support an inhibitory effect of phosphorylated MEPE-derived ASARM peptide on the formation of mineralized dentin. In this model, a reparative dentin bridge normally forms following injury and operates as a barrier to protect the dental pulp [39], [47]. We previously reported abnormal dentin mineralization in the proximity of p-ASARM peptide soaked beads using this rat model [12]. Here we show in additional studies that only the phosphorylated form of the ASARM peptide impairs mineralization, and that immunohistochemistry for MEPE shows more intense staining of odontoblasts adjacent to the reparative dentin bridge after p-ASARM treatment than after np-ASARM treatment as applied by peptide-soaked agarose bead placement at the wound site. Moreover, here we have quantified pulp volume to show that it is significantly higher in rat molars treated with inhibitory p-ASARM, indicating decreased mineralization relative to controls. Paralleling this, when compared to age-matched control patients with normal teeth, we previously reported a 2-fold higher pulp ratio (pulp chamber area/tooth area) in deciduous molars of children with XLH, and a 1.6-fold higher pulp ratio in permanent teeth of young adults with XLH [3].

**Figure 7:**
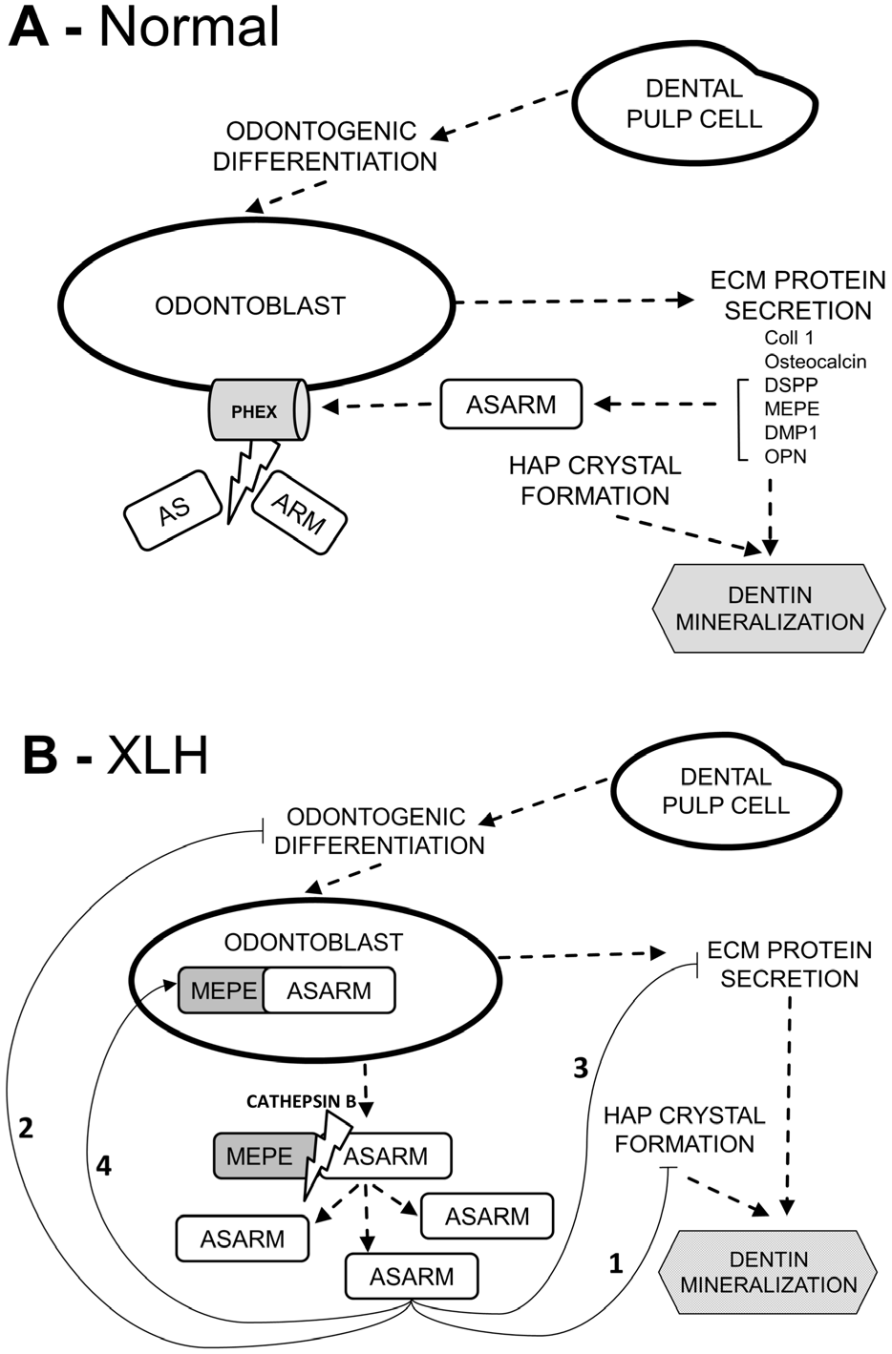
Summary of the role of the MEPE-derived ASARM peptide in the etiology of tooth dentin abnormalities in XLH patients. A: SIBLING proteins containing the ASARM peptide are processed by a multitude of proteolytic enzymes, some of which may release the ASARM peptide or larger protein fragments containing the ASARM peptide into the extracellular matrix (ECM). ASARM and ASARM-containing peptides are inhibitory for mineralization, binding directly to hydroxyapatite (HAP) mineral crystals in bones and teeth. In normal conditions, neutralizing PHEX cleavage of ASARM releases extracellular matrices from this inhibition and mineralization proceeds appropriately. B: In XLH tooth dentin, inactivating mutations in the *PHEX* gene result in nonfunctional PHEX enzyme that allows HAP crystal-binding, ASARM-containing peptides to accumulate in the dentin thus inhibiting tooth mineralization (pathway 1). Normal PHEX also protects full-length MEPE from cleavage by proteases (cathepsin B), thereby preventing release of mineralization-inhibiting ASARM. In XLH, excessive cleavage of MEPE by proteases (in the absence of functional PHEX) to release the inhibitory ASARM peptide might also contribute to the impaired mineralization of dentin. Finally, ASARM accumulation in XLH may impair dentinogenesis by decreasing odontoblast differentiation and downregulating genes encoding for secreted ECM proteins (pathways 2 and 3), while increasing MEPE expression (pathway 4) which in turn would further exacerbate the XLH hypomineralization tooth phenotype.

The cleavage and protein-protein interactions of MEPE have been implicated in the pathogenicity of XLH [23]. Notably, it has been reported that MEPE is downregulated as odontoblasts differentiate [65], [66]. In our *in vitro* experiments, p-ASARM significantly increased MEPE expression, both at the mRNA and protein levels. In addition, odontoblast-like cells producing reparative bridge dentin in rat dental pulps implanted with p-ASARM peptides strongly expressed MEPE. Thus, our *in vitro* and *in vivo* results indicate that the p-ASARM peptide enhances MEPE expression, which in turn might delay pulp stem cell differentiation as might be expected to occur in XLH patients. Moreover, accumulation of MEPE in the unmineralized heterogeneous dentin bridge regions identified in p-ASARM-treated rats is consistent with our previous observation showing that MEPE accumulates in the unmineralized interglobular spaces (between calcospherites) of dentin in patients with XLH [13]. Furthermore, the increased expression of MEPE we observed in the presence of p-ASARM both in the 3D culture model and in the wounded pulp rat model is consistent with previous publications reporting an upregulation of MEPE in osteoblasts of Hyp mice [16], [20], [23], [37]. Thus, in XLH, overexpressed MEPE in osteoblasts (and shown here now in odontoblasts) results in the potential for more ASARM peptide to accumulate in the ECM of bone and dentin, which would not be cleaved away (as would normally occur by the enzymatic activity of PHEX) as a result of the inactivating PHEX mutations (summarized in Fig. 7). As the ASARM peptides are highly resistant to proteases and are only known to be degraded by PHEX [23], [33], this positive-feedback loop on MEPE expression induced by ASARM peptides might exacerbate the disease and would thus be a mechanism contributing in part to the mineralization defect seen in XLH. Indeed, increased levels of ASARM peptide in serum have been reported in hypophosphatemic patients and in Hyp mice [31], [61]. Furthermore, the ASARM peptide has been shown to accumulate in the renal tubules of Hyp mice [61], and recent evidence suggests that it may directly impair phosphate uptake and induce hypophosphatemia [31]. Studies are currently in progress using pulp cells derived from XLH patients to better understand the function of PHEX in this complex pathologic process.

In conclusion, using *in vitro* and *in vivo* models we demonstrate that the MEPE-derived ASARM peptide – previously identified in XLH dentin – inhibits both ECM mineralization and odontoblast differentiation, while increasing MEPE expression. These results provide for a partial mechanistic explanation of the pathogenesis of XLH whereby direct inhibition of mineralization by p-ASARM positively reinforced by increased MEPE expression induced by ASARM, together with decreased odontoblast differentiation to produce less dentin, leads to the mineralization defect and increased pulp chamber characteristic of teeth in XLH. Further studies are required to determine whether the MEPE-derived ASARM peptide affects odontoblast differentiation through indirect cell-signaling induced by defective ECM mineralization and/or by direct cell signaling with potential internalization of the peptide by cells. Collectively, these data appear promising in guiding the development of novel therapies for XLH through targeting the MEPE-ASARM system.

## Supporting Information

Figure S1 **Polychromatic flow cytometry analysis of dental pulp cells from deciduous teeth.** More than 95% of cells at passage 2 were CD45-. In addition, most of them were CD90+/CD29+/CD44+/CD146+/CD105+/CD73+. About 10% were STRO-1+.
(TIFF)

Figure S2 **Interaction of cells with the tooth dentin slice.** Scanning electron microscopy of an interaction of SHEDs with the tooth dentin slice at day 21. Images show a close relationship between the cell processes (asterisks) and the dentin surface (containing many dentinal tubules). Higher magnification of the white dashed frame is shown in the right panel.
(TIFF)

Figure S3 **MEPE-ASARM peptides do not affect PHEX expression.** SHED cell cultures were maintained in nonmineralizing (NM) or mineralizing (M) conditions in the absence or presence of 20 µM of either phosphorylated (p-ASARM) or nonphosphorylated (np-ASARM) ASARM peptide for 21 days. Immunofluorescence microscopy (left panel) and Western blotting (right panel) were performed at day 21. Immunofluorescent staining for PHEX (arrows) is observed in the SHEDs under all conditions. Western blot analysis shows similar levels of PHEX protein without any significant differences between the different conditions, when normalized to cellular tubulin content.
(TIFF)
