# Supplementary figures and images for: MEPE-Derived ASARM Peptide Inhibits Odontogenic Differentiation of Dental Pulp Stem Cells and Impairs Mineralization in Tooth Models of X-Linked Hypophosphatemia
Source: PLoS One. 2013 Feb 22;8(2):e56749. doi: 10.1371/journal.pone.0056749 (PMC3579870; doi:10.1371/journal.pone.0056749)

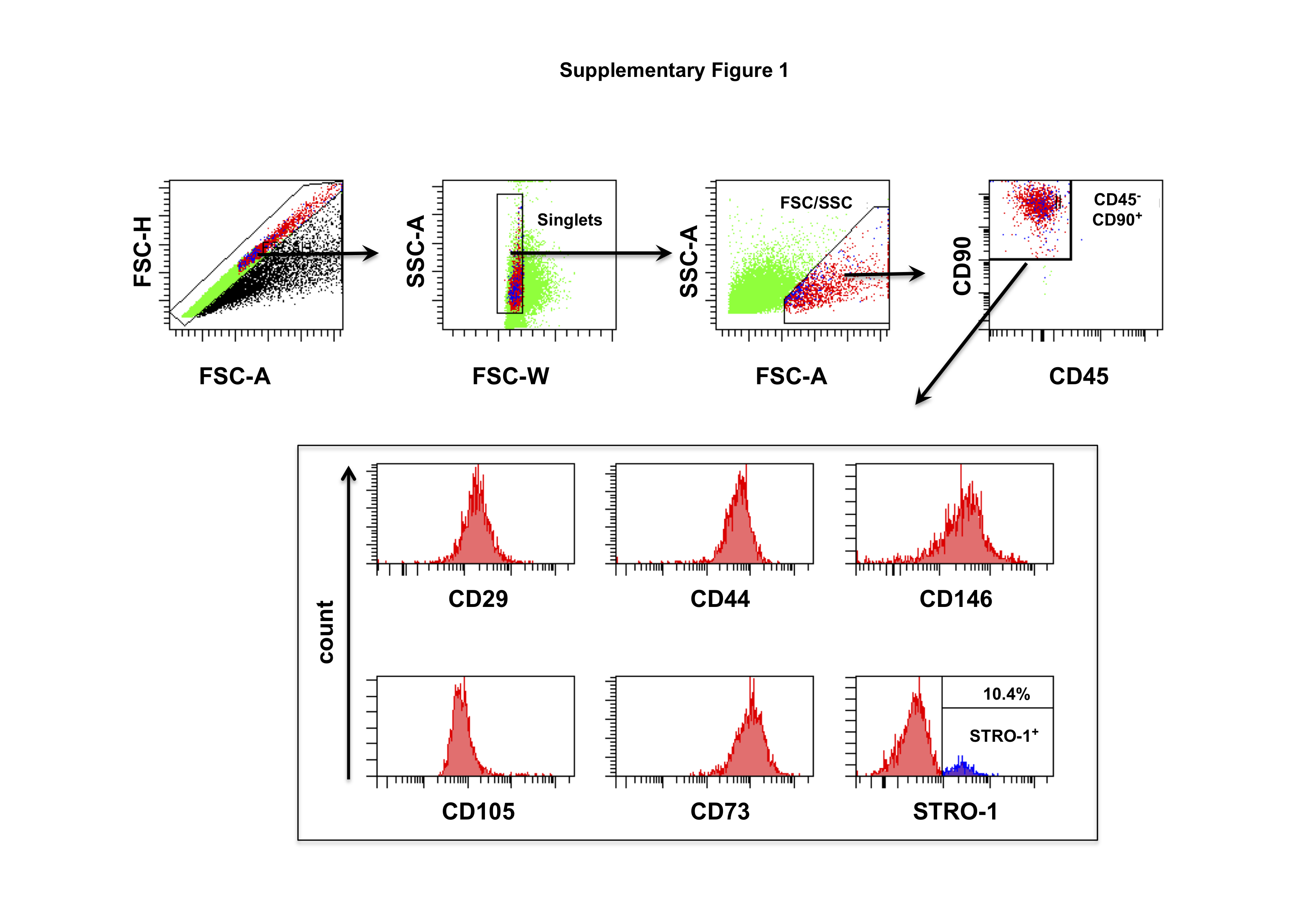

Supplement: Figure S1 — Polychromatic flow cytometry analysis of dental pulp cells from deciduous teeth. More than 95% of cells at passage 2 were CD45-. In addition, most of them were CD90+/CD29+/CD44+/CD146+/CD105+/CD73+. About 10% were STRO-1+. (TIFF) [file pone.0056749.s001.tif]

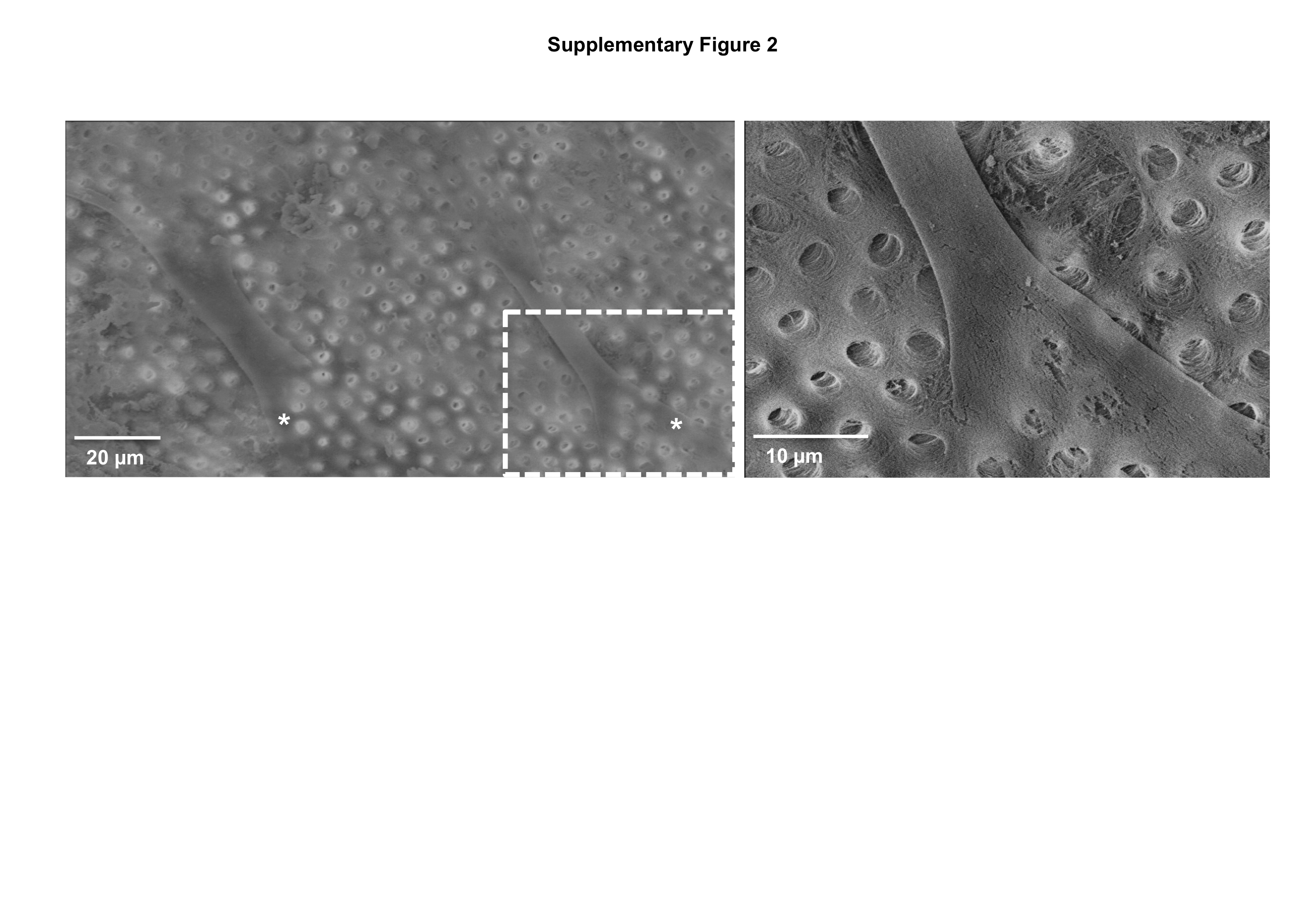

Supplement: Figure S2 — Interaction of cells with the tooth dentin slice. Scanning electron microscopy of an interaction of SHEDs with the tooth dentin slice at day 21. Images show a close relationship between the cell processes (asterisks) and the dentin surface (containing many dentinal tubules). Higher magnification of the white dashed frame is shown in the right panel. (TIFF) [file pone.0056749.s002.tif]

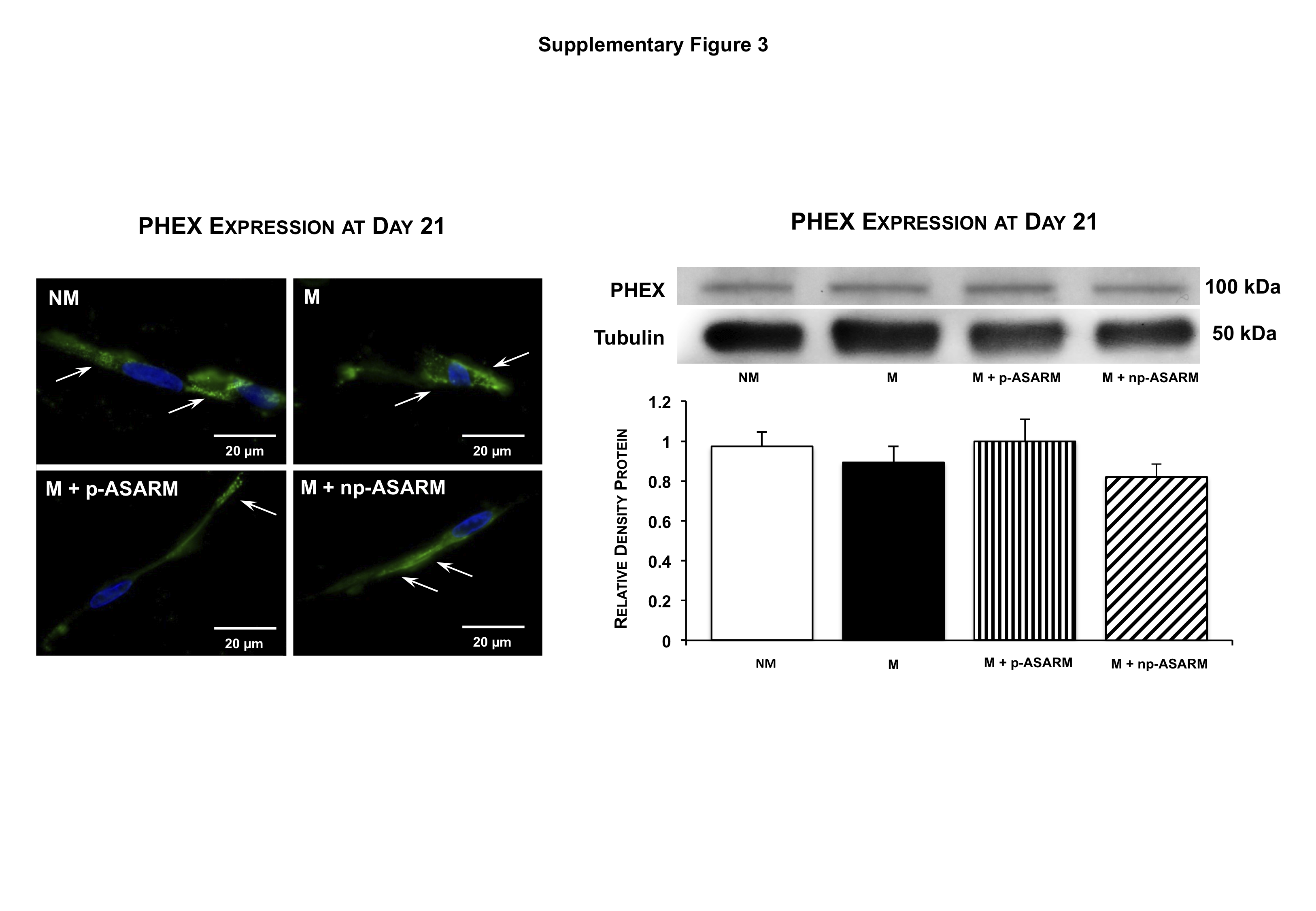

Supplement: Figure S3 — MEPE-ASARM peptides do not affect PHEX expression. SHED cell cultures were maintained in nonmineralizing (NM) or mineralizing (M) conditions in the absence or presence of 20 µM of either phosphorylated (p-ASARM) or nonphosphorylated (np-ASARM) ASARM peptide for 21 days. Immunofluorescence microscopy (left panel) and Western blotting (right panel) were performed at day 21. Immunofluorescent staining for PHEX (arrows) is observed in the SHEDs under all conditions. Western blot analysis shows similar levels of PHEX protein without any significant differences between the different conditions, when normalized to cellular tubulin content. (TIFF) [file pone.0056749.s003.tif]
